# Ongoing removals of invasive lionfish in Honduras and their effect on native Caribbean prey fishes

**DOI:** 10.7717/peerj.3818

**Published:** 2017-10-18

**Authors:** Friederike Peiffer, Sonia Bejarano, Giacomo Palavicini de Witte, Christian Wild

**Affiliations:** 1 Coral Reef Ecology Group, Marine Ecology Department, Faculty of Biology and Chemistry, University of Bremen, Bremen, Germany; 2 Department of Ecology, Leibniz Centre for Tropical Marine Research (ZMT), Bremen, Germany; 3 Roatan Marine Park, Roatan, Islas De La Bahia, Honduras; 4 Current affiliation: Shark Legacy Project, Roatan, Islas De La Bahia, Honduras

**Keywords:** Invasive lionfish, Population control, Removal effort, Native prey fish, Roatan, Caribbean

## Abstract

The invasion of Indo-Pacific lionfish is one of the most pressing concerns in the context of coral reef conservation throughout the Caribbean. Invasive lionfish threaten Caribbean fish communities by feeding on a wide range of native prey species, some of which have high ecological and economic value. In Roatan (Honduras) a local non-governmental organisation (i.e. Roatan Marine Park) trains residents and tourists in the use of spears to remove invasive lionfish. Here, we assess the effectiveness of local removal efforts in reducing lionfish populations. We ask whether reefs subject to relatively frequent removals support more diverse and abundant native fish assemblages compared to sites were no removals take place. Lionfish biomass, as well as density and diversity of native prey species were quantified on reefs subject to regular and no removal efforts. Reefs subject to regular lionfish removals (two to three removals month^−1^) with a mean catch per unit effort of 2.76 ± 1.72 lionfish fisher^−1^ h^−1^ had 95% lower lionfish biomass compared to non-removal sites. Sites subject to lionfish removals supported 30% higher densities of native prey-sized fishes compared to sites subject to no removal efforts. We found no evidence that species richness and diversity of native fish communities differ between removal and non-removal sites. We conclude that opportunistic voluntary removals are an effective management intervention to reduce lionfish populations locally and might alleviate negative impacts of lionfish predation. We recommend that local management and the diving industry cooperate to cost-effectively extend the spatial scale at which removal regimes are currently sustained.

## Introduction

Biological invasions are a serious threat to ecosystem biodiversity and function (Lowe et al., 2000). Invasion patterns and processes are well known in terrestrial ecosystems whereas marine invaders are far more common and occur in all marine ecoregions (Molnar et al., 2008). Invasive species in both marine and terrestrial ecosystems usually occupy low trophic levels (e.g. algae, invertebrates) (Byrnes, Reynolds & Stachowicz, 2007; Côté, Green & Hixon, 2013). The invasion of the Caribbean sea by lionfish native to the Indo Pacific is a rare example of predatory fish invading coral reefs at a rate and magnitude previously undocumented in any marine system (Côté, Green & Hixon, 2013). While the first lionfish was reported in 1985 off the coast of Florida, the invasion throughout the Caribbean started in the early 2000’s when they invaded the Bahamas (Schofield, 2009). A decade later, lionfish were established throughout the Caribbean becoming one of the most pressing conservation concerns throughout the region (Gómez Lozano et al., 2013).

Concerns stem from the fast pace and spatial extent of the invasion, but also from the serious ecological consequences associated to the voracious predatory behaviour of lionfish (Albins & Hixon, 2008) and their interaction with other native predators (Hackerott et al., 2013; Ellis & Faletti, 2016). Naïveté to invasive lionfish has been documented in at least eight native prey fishes, and plays an important role in the high consumption rates of these exotic predators. (Anton et al., 2016). Lionfish diets throughout the invaded range are broader and include larger prey compared to their native range (Cure et al., 2012). Predation rates of *Pterois volitans* in the Caribbean are also considerably higher than those recorded for native counterparts (Côté & Maljkovic, 2010; Green, Akins & Côté, 2011). Up to 60 native fish species and many invertebrate species (e.g. crustaceans, molluscs) have been found in lionfish stomach contents in the Caribbean (Morris & Akins, 2009; Côté & Maljkovic, 2010; Valdez-Moreno et al., 2012; Côté et al., 2013). Importantly, lionfish exert 2.5 ± 0.5 times higher prey consumption rates compared to a similar-sized native predator, the coney grouper *Cephalopholis fulva* (Albins, 2013). As a result, lionfish in the invaded range grow larger and heavier, and are more abundant than in their native range (Darling et al., 2011; Pusack et al., 2016). Consequently lionfish reduce abundance and species richness of prey sized reef fishes (Albins, 2015; Benkwitt, 2015), affecting also ecological important herbivorous fish (Albins, 2015). Following the lionfish invasion in the Bahamas, average biomass of native prey species declined by 65% in two years (Green et al., 2012). Small demersal fishes with shallow bodies were particularly vulnerable to lionfish predation (Green & Côté, 2014). Recent evidence indicates that lionfish have reduced the abundance of tomtates (*Haemulon aurolineatum)* off the southeastern United States (North Carolina to Florida) by 45% since the onset of the invasion (Ballew et al., 2016). Similarly, in a controlled field experiment lionfish caused increased mortality rates and drove local extirpation of fairy basslets (*Gramma loreto*) (Ingeman, 2016). In a possible worst-case scenario, the invasion could result in losses of biodiversity and abundance of important fish species, negatively affecting coral reef ecosystem function, weakening ecosystem services, and threatening livelihoods within coastal communities (Albins & Hixon, 2011).

In response to these concerns, management efforts have focused on controlling populations to levels that could minimise ecological and economic impacts (Gómez Lozano et al., 2013). Targeted spearfishing emerged in 2010 as a relatively simple and effective control measure and is now widely practised throughout the region (Akins, 2012). Although eliminating lionfish completely is unrealistic, removal efforts could help maintain low lionfish abundances (Morris & Whitfield, 2009; Albins & Hixon, 2011). Reduced numbers of lionfish could release native prey from predation, and therefore conserve a more diverse and abundant assemblage of native prey sized-fishes. In contrast, on reefs subject to no lionfish removals, high lionfish abundance and predation rates may result in assemblages with lower densities, biomass and species richness of prey-sized native species (Green et al., 2012; Albins, 2013, 2015; Benkwitt, 2015).

Currently, campaigns to extract lionfish using scuba-based spearfishing are well anchored in many national management plans. Efforts to determine the extent to which management efforts can effectively reduce lionfish populations remain however rare in the wider Caribbean (but see Frazer et al., 2012; León et al., 2013). In Little Cayman, for instance, targeted removals maintained lionfish biomasses below 12–133 fish ha^−1^ over >70 days (Frazer et al., 2012). In Bonaire and Curacao, removals lead to a 2.76-fold reduction of lionfish biomass on fished compared to unfished sites (León et al., 2013). As the effectiveness of lionfish removal programmes depends on the pre-invasion characteristics of native fish assemblage, and the duration of exposure to the invasion (Green et al., 2014), it can be spatially heterogeneous. It is therefore important to expand the spatial coverage of studies assessing the effectiveness of removals and associated ecological benefits to different areas in the Caribbean. Importantly, financial resources limit the intensity and geographical scope of removal programmes. Effectiveness of targeted removals vary according to the degree of organisation of the removal program, the amount of people involved, and the frequency and continuity of fishing effort invested (Bayraktarov et al., 2014). Maintaining regular removals over reasonably high spatial scales may therefore be more feasible in wealthy nations, or where effective cooperation among non-governmental organisations (NGOs), diving industry and national government exists. In developing nations with fewer resources (e.g. Honduras) little is known about the level of organisation and consistency of removal efforts. Gathering information in Honduras is important from a local perspective because control efforts are critical for mitigating the effects of lionfish on key marine habitats such as marine protected areas (Morris, 2012). From a regional perspective, Honduras is the country with the highest lionfish densities in the Mesoamerican barrier reef region (McField, 2012) and may act as a source of larvae for re-colonization of unmanaged areas nearby. Therefore, lionfish population size and spatial extent and frequency of removals around the region will likely affect removal effectiveness elsewhere.

Here, we investigated the practice of lionfish removals inside a marine reserve in Roatan (Honduras), where little is known about its extent and effectiveness. We started by identifying the sectors of the public engaged in lionfish removals, and the reefs where removal efforts concentrate. We then quantified the frequency of removal trips, as well as the catch per unit effort (CPUE) per trip over six months. Specifically, we asked whether reefs subject to relatively regular removals sustain lower lionfish biomass and more diverse and abundant native prey fish assemblages, compared to sites were no removals take place. The ultimate goal of this study was to expand the knowledge on the effectiveness of organised lionfish removal programmes towards the Western Caribbean, an area still poorly studied for the impact of lionfish.

## Materials and Methods

### Study area

**Figure 1:**
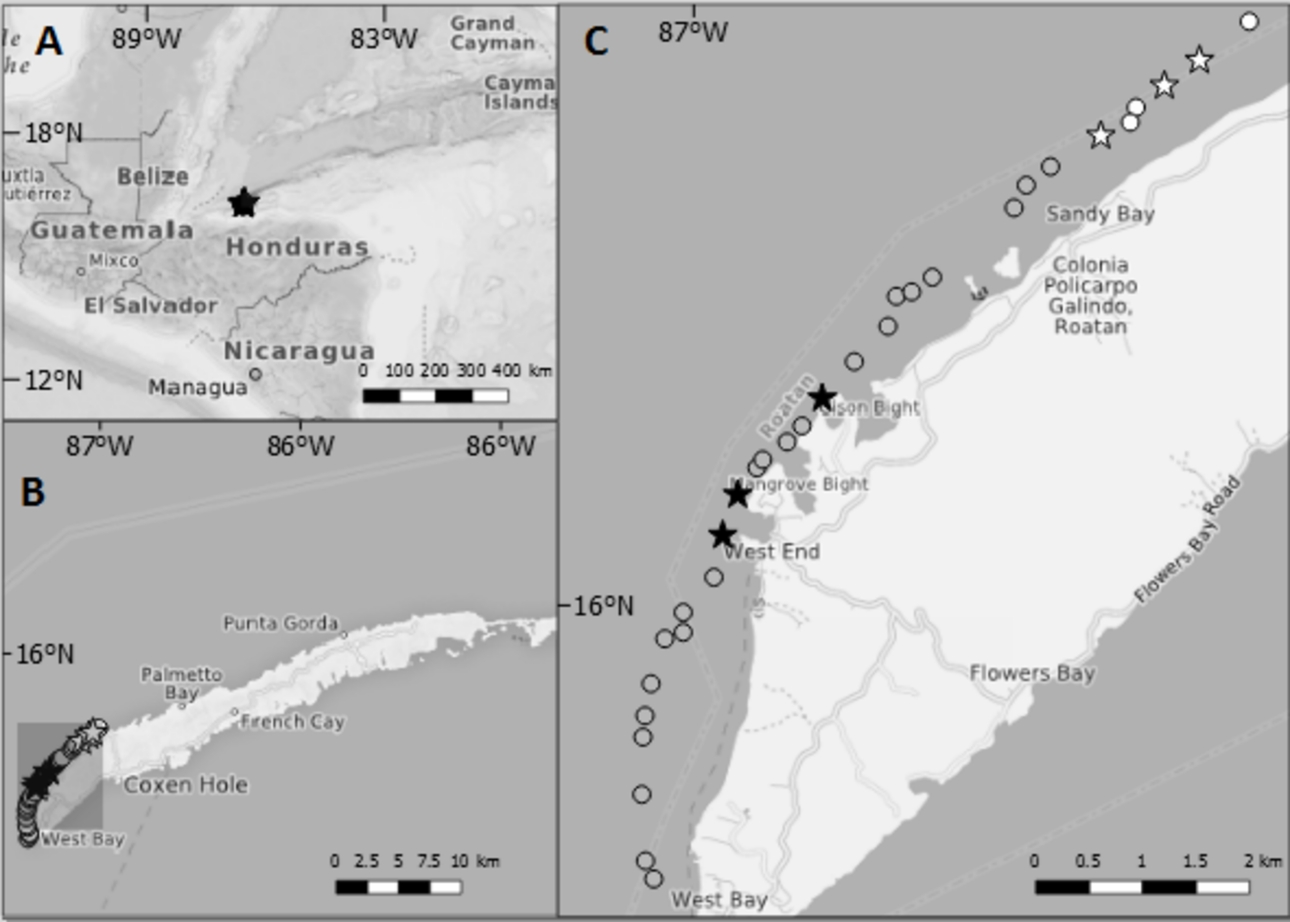
Maps showing the location of Roatan and the study sites inside Sandy Bay-West End Marine Reserve. (A) Location of Roatan relative to Central America. (B) The island of Roatan which is part of the Bay Islands, Honduras. (C) Each point gives the location of dive sites along northwest coast of Roatan. Stars indicate dive sites at which data on lionfish and native fish assemblages were collected for this study. Black shading = sites where lionfish were culled at least once per month; grey shading = sites where lionfish were culled less than once per month; white = sites where culling was never reported. The former lay in the proximity of the villages West End and West Bay, the villages with the highest number of dive operators, whereas the latter lay close to the border of the Marine Reserve and ~3 km away from West End.

This study was conducted on the island of Roatan, which is part of the Bay Islands, located approximately 60 km north off the coast of Honduras in the Western Caribbean (Fig. 1). The coral reefs surrounding the island are part of the Mesoamerican Barrier Reef System. Invasive lionfish were first reported in Roatan in 2009, after which Honduras became the country with the highest lionfish densities in the Mesoamerican barrier reef region (McField, 2012). In 2010, a local non-governmental organisation (Roatan Marine Park) received permission from the governing agency for fisheries in Honduras (DIGIPESCA) to distribute pole spears among the island residents. These were to be used exclusively on lionfish to aid in controlling the invasion. The first lionfish derby was hosted by Roatan Marine Park in 2011, during which 1,200 lionfish were removed. Little else is known about the frequency and extent of organised removal efforts.

Data for this study were collected during a single field campaign (October 2014–March 2015) with three main components: (a) stakeholder interviews and site selection based on the spatial distribution of the lionfish removal efforts (October–December 2014), (b) surveys of removal frequency and CPUE (October 2014–March 2015), and (c) underwater surveys of invasive lionfish and native prey fishes (February 2015) and benthic attributes of the reefs (March 2015). For this study, we chose dive sites that differed in lionfish removal effort but were otherwise visually similar in habitat characteristics.

Based on initial stakeholder interviews, six study sites were selected from which three sites were subject to regular lionfish removal efforts and three sites were subject to no lionfish removals during the study period. All sites were located on the forereef slope of a fringing reef located 300 m from the shore, within the Sandy Bay-West End Marine Reserve on the northwest side of the island (Fig. 1). Commercial fishing and fishing on herbivorous fish is prohibited within the reserve, and the regulation is enforced by a regular patrol by rangers of Roatan Marine Park. Underwater surveys were conducted at 15 m water depth at the start of a steep reef wall that ends approximately at 30 m water depth dropping into a sandy bottom and followed by the Cayman Trench (Gonzalez, 2013).

### Field surveys

#### Stakeholder interviews and spatial extent of the removal efforts

During the first four weeks of the study, interviews were conducted with relevant authorities and stakeholders on the Island including subsistence fishers, dive and tourism operators, and NGOs. To obtain information on the spatial distribution of removals, interviewees were asked to provide information on whether they conducted regular removals and where. We started by interviewing people working at Roatan Marine Park (three people) who already had a good overview on who is fishing where. Based on their knowledge, we interviewed mostly people from the dive and tourism operations (∼20 people) to identify sites where lionfish removals were most frequent. As not many subsistence fishers reside in the Sandy Bay-West End Marine Reserve area, we interviewed only five of them who claim to regularly fish elsewhere and only occasionally catch and sell lionfish. Based on interview responses, we established that (a) catch surveys would focus on removals coordinated by dive operators and Roatan Marine Park as no other sector of the public was significantly engaged in the removal efforts, and (b) the Sandy Bay-West End Marine Reserve would be the focus area of our study because it was the most frequented diving area. The three most popular dive sites among dive operators were selected as removal sites and three comparable reef sites that were not visited by dive operators for lionfish removals were selected as non-removal sites. Sites were separated by at least 300 m from each other, and treatments were at least 2 km apart from non-removal sites.

#### Frequency of lionfish removals and CPUE

Printed questionnaires were distributed fortnightly for the entire duration of the study among dive operators in the West End/West Bay area. Questionnaires were answered per diver and included the location of the dive, total dive time, maximum water depth and the number of lionfishes successfully speared. This information was used to measure removal frequency and the CPUE. The CPUE was calculated as the number of lionfish removed by divers divided by the sum of their bottom time in hours (Frazer et al., 2012).

#### Underwater visual censuses of lionfish and native prey fishes

Lionfish biomass, a proxy for the effectiveness of targeted removals, was quantified using underwater visual censuses at the selected sites during on-going lionfish removals (Fig. 1). Visual censuses were conducted along 50 × 5 m (0.025 ha) transects, and three replicate transects were surveyed per site in February 2015. All transects were laid out at 15 m water depth, parallel to the reef crest, and separated at least 10 m from each other. All transects were inspected by the same surveyor swimming in a zigzag pattern at a moderate and constant speed, while thoroughly examining overhangs and crevices to ensure the detection of cryptic lionfish (Morris, 2012; Green et al., 2013). Alongside, a second diver counted larger species of snapper and grouper (species list: Table S1), which are native predators of smaller fishes, within the same transect. Simultaneously, the total length (TL) of lionfish and native predators were estimated by the divers. To avoid diurnal–nocturnal changeover periods of fish, all censuses were done during daylight hours.

Density of prey fishes (<15 cm) (species list: Table S2) was quantified using underwater visual censuses along 25 × 2 m transects (0.005 ha) in February 2015. Three replicate transects were surveyed per site, by a single diver recording the species, number, and size classes (<5, <10 and <15 cm) of all native fishes with less than 15 cm in size. Care was taken to inspect cryptic spaces. Due to dive time constraints reliable counts of gobies (Gobiidae) and blennies (Blennidae) could not be included.

#### Benthic attributes of survey sites

To account for benthic characteristics that may influence reef fish diversity and abundance, we quantified the percent cover of 12 major benthic categories (i.e. hard coral, soft coral, dead coral, fire coral, gorgonian, sponge, macro-algae, turf-algae, crustose coralline algae, cyanobacteria, sand and rubble) as well as topographic complexity. Benthic cover was measured along three 50 m point-intercept transects (PIT) per site according to the method by Hill & Wilkinson (2004). Substratum height was recorded as a proxy for topographic complexity, every 5 m along the PITs by measuring the distance between lowest and highest point of the reef within a 1 m radius of the transect tape (Morris, 2012).

### Data analysis

All statistical analyses were conducted by fitting linear mixed-effects models with the function lme in the nlme package in R (Pinheiro et al., 2014). Differences between removal and non-removal sites were assessed by using analysis of variance (ANOVA) (Zuur et al., 2009). The adequacy of the models was tested by (1) plotting the Pearson residuals against the fitted values and against each explanatory variable in the model to check for homogeneity and (2) the normality Q–Q plot to test for normality of errors (Zuur, Ieno & Smith, 2007). The model used to investigate the differences in biomass of native predators between removal and non-removal sites required a variance structure (i.e. varIdent) to account for heteroscedasticity, and allow for different variances per treatment group (Zuur et al., 2009). We first run models aimed to discard that habitat characteristics that could influence lionfish or native prey abundance were different between removal and non-removal sites. Second, we run models to ask whether lionfish biomass and prey density differed between removal and non-removal sites.

#### Accounting for confounding factors that may have affected lionfish biomass and prey density

Habitat characteristics and biomass of native predators at the sites could influence lionfish biomass and prey fish density. We therefore selected sites that had visually similar benthic communities and topographic complexity but also corroborated that these did not differ significantly between removal and non-removal sites. Hard coral cover, topographic complexity and native predator biomass were therefore modelled as a function of removal treatment.

#### Effectiveness of lionfish removals

To investigate whether sites subject to removals have significantly lower lionfish biomass (kg ha^−1^) compared to non-removal sites, linear mixed-effects models were used. Transect-level data of lionfish biomass was modelled as a function of removal treatment as a fixed effect. Site was included as a random effect to account for random variability across sites.

#### Are lionfish removals associated with diverse and abundant prey fish assemblages?

Linear mixed-effects models were also used to investigate whether sites subject to removals sustain more diverse and abundant native prey assemblages compared to non-removal sites. Transect-level data of prey fish density (individuals ha^−1^), as well as site-level data of taxonomic diversity (i.e. Shannon–Wiener index H) and richness were modelled as a function of removal treatment (fixed effect) and site as random effect.

## Results

### Stakeholder interviews and spatial extent of the removal efforts

Targeted lionfish removals were largely sustained by the recreational dive industry which is responsible for 78% of the monthly total catch in the marine reserve, whereby tourists contributed with 13% of the catch. A few local residents outside of the diving industry were involved in regular culling efforts, contributing to 22% to the total catch per month. As a result, the spatial distribution of removals was dictated merely by the popularity and accessibility of reefs from a recreational diving perspective. The most popular dive sites located within 3 km of the dive operators were also the sites with highest removal frequency.

### Frequency of lionfish removals and CPUE

From 15th of September 2014 to 15th of March 2015 a total of 482 lionfish were removed at approximately 80% of the dive sites on the north side of the Sandy Bay-West End Marine Reserve. During this time, 29 divers with different levels of experience and degree of contribution to the overall catch were involved in lionfish removals. Removals spanned a water depth range of 5–45 m, and lionfish in the catches ranged between 5–39 cm in TL (21.76 ± 9.22; mean ± SE). At our regularly fished study sites, the mean frequency of removals was two to three times a month with an average CPUE of 2.76 ± 1.72 (mean ± SE) lionfish fisher^−1^ h^−1^.

### Confounding factors that may have affected lionfish biomass and prey density

**Figure 2:**
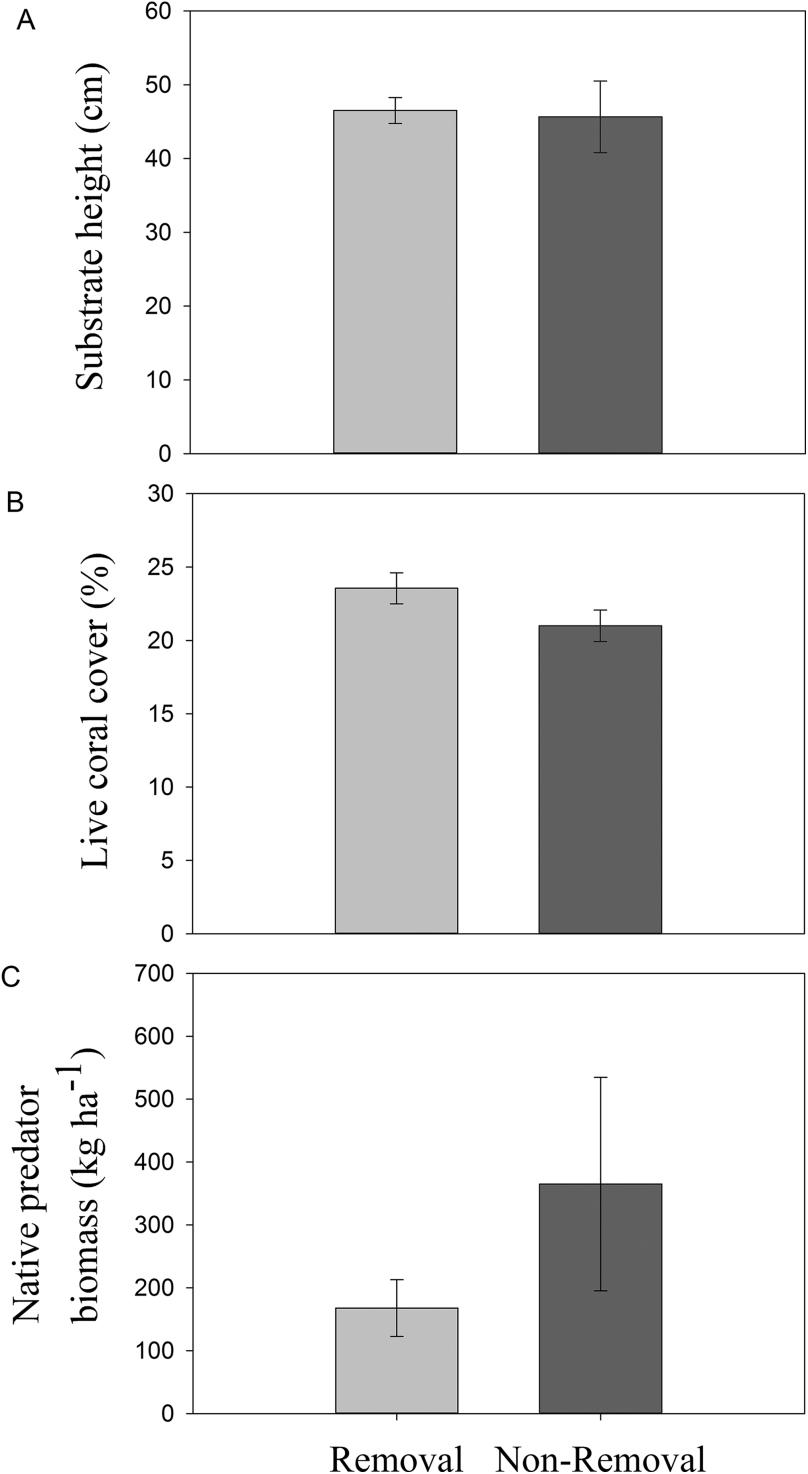
Habitat characteristics of removal and non-removal sites. (A) topographic complexity as substratum height, (B) live coral cover and (C) native predator biomass in removal and non-removal sites. No statistically significant differences were detected between removal and non-removal sites in all cases (topographic complexity: *p*_ANOVA_ = 0.88; live coral cover: *p*_ANOVA_ = 0.17; native predator biomass: *p*_ANOVA_ = 0.27, Table 1).

No significant differences in live coral cover (*p*_ANOVA_ = 0.16), topographic complexity (*p*_ANOVA_ = 0.88), or native predator biomass (*p*_ANOVA_ = 0.27) were observed between sites subject to removals and controls without removals (Table 1; Fig. 2).

**Table 1:** Model parameters and significance values indicating relationships between lionfish removals and lionfish biomass, prey fish density and confounding factors.

*Accounting for confounding factors that may influence fish communities*				
1.) *Hard coral cover*				
Model terms	numDF	denDF	*F*-value	*p*-value
(Intercept)	1	12	873.9185	<.0001
Removal	1	4	2.8750	0.1652
2.) *Substratum height*				
Model terms	numDF	denDF	*F*-value	*p*-value
(Intercept)	1	12	318.9928	<.0001
Removal	1	4	0.0277	0.8759
3.) *Native predator biomass*				
Model terms	numDF	denDF	*F*-value	*p*-value
(Intercept)	1	12	16.090918	0.0017
Removal	1	4	1.655994	0.2676
*Lionfish biomass as function of lionfish removal*				
Model terms	numDF	denDF	*F*-value	*p*-value
(Intercept)	1	12	71.79794	<.0001
Removal	1	4	59.08971	0.0015*
*Prey fish density as function of lionfish removal*				
Model terms	numDF	denDF	*F*-value	*p*-value
(Intercept)	1	12	540.7013	<.0001
Removal	1	4	23.5600	0.0083*
*Prey fish species richness as function of lionfish removal*				
Model terms	numDF	denDF	*F*-value	*p*-value
(Intercept)	1	4	338.56	0.0001
Removal	1	4	0.64	0.4685
*Prey fish taxonomic diversity as function of lionfish removal*				
Model terms	numDF	denDF	*F*-value	*p*-value
(Intercept)	1	4	1040.4761	<.0001
Removal	1	4	1.1986	0.3351

**Note:**

The codes ‘numDF’ and ‘denDF’ indicate the numerator degrees of freedom and the denominator degrees of freedom.

### Effectiveness of lionfish removals

**Figure 3:**
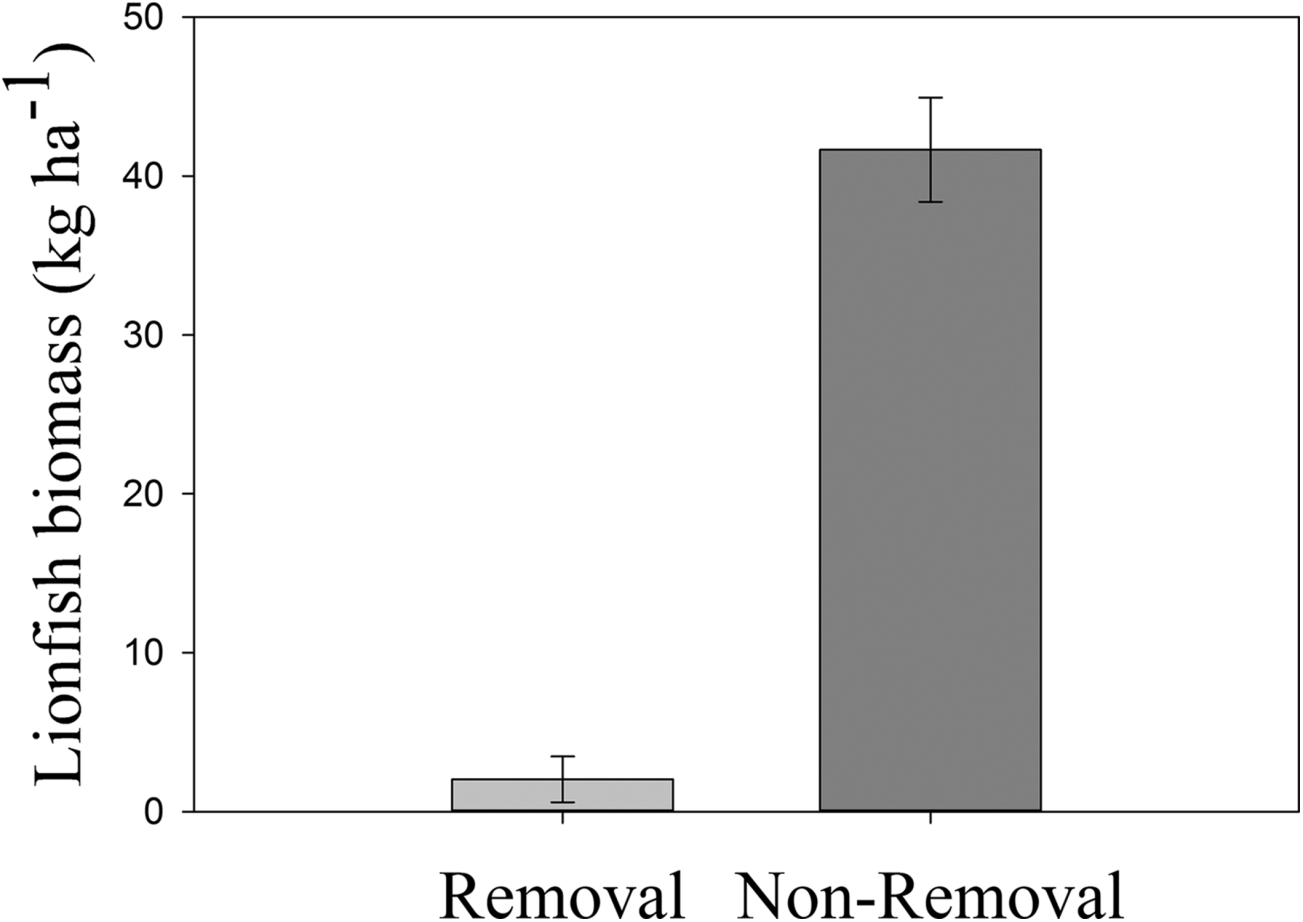
Mean lionfish biomass in removal and non-removal sites. Significant differences in mean (±SE) lionfish biomass (kg ha^−1^) were detected between removal and non-removal sites in Roatan (*p*_ANOVA_ = 0.0015, Table 1).

Lionfish biomass was significantly lower in removal compared to non-removal sites (*p*_ANOVA_ = 0.0015, Table 1; Fig. 3). Mean lionfish biomass was 20 times lower at removal (2.02 ± 1.44 kg ha^−1^) compared to non-removal sites (41.66 ± 3.28 kg ha^−1^, Fig. 3). The average body size at removal sites was lower (25.00 ± 5.00 cm) than at non-removal sites (32.24 ± 0.96 cm). However, the standard error is high due to a low sample size at removal sites (*n* = 2).

### Are lionfish removals associated with diverse and abundant prey fish assemblages?

**Figure 4:**
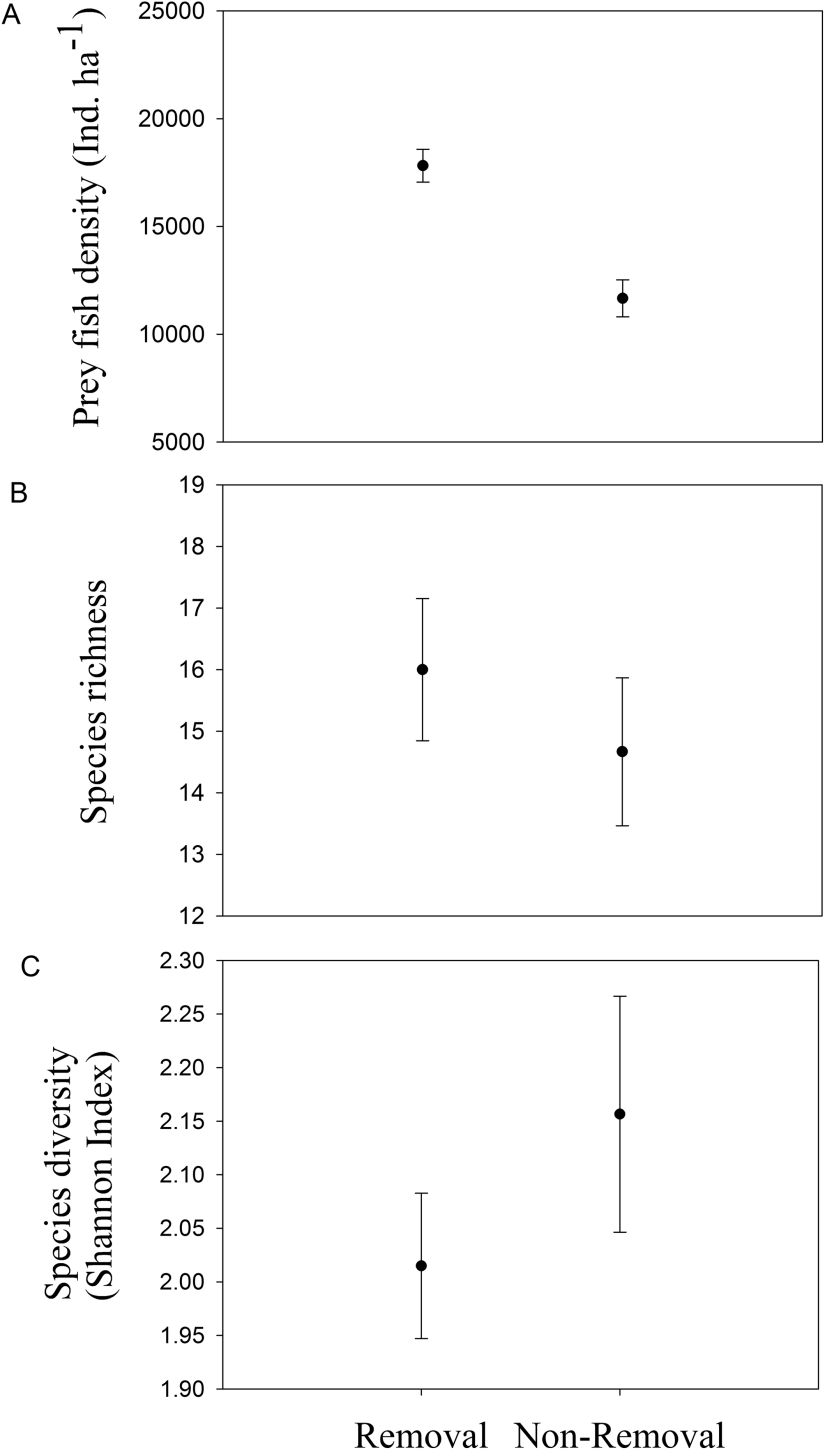
Native prey fish in removal and non-removal sites. (A) density, (B) species richness and (C) taxonomic diversity (Shannon–Wiener Index) of native prey fish at removal and non-removal sites. Density of prey fish was significantly lower on removal compared to non-removal sites (*p*_ANOVA_ = 0.008, Table 1) whereas species richness and diversity did not significantly differ between sites (species richness: *p*_ANOVA_ = 0.4685, Table 1 and species diversity: *p*_ANOVA_ = 0.34, Table 1).

Mean density of native fishes was significantly lower in removal sites compared to non-removal sites (*p*_ANOVA_ = 0.008, Table 1; Fig. 4A). Neither native prey species richness nor taxonomic diversity was significantly different between removal and non-removal sites (species richness: *p*_ANOVA_ = 0.4685, Table 1; Fig. 4B and species diversity: *p*_ANOVA_ = 0.34, Table 1; Fig. 4C).

## Discussion

While lionfish removal efforts are broadly popular in the Caribbean, the extent to which these are embedded in community activities and management programmes, as well as their effectiveness and expected benefits may vary widely among countries. We find that lionfish removals inside the Sandy Bay-West End Marine Reserve in Roatan are voluntary and opportunistic in nature, and are almost exclusively sustained by recreational dive operators. On dive sites subject to two to three removal visits per month and a CPUE of 2.76 ± 1.72 lionfish fisher^−1^ h^−1^, lionfish biomass was 20 times lower compared to similar sites where no removals occur. Higher densities of native prey fishes were found on non-removal compared to removal sites, but the corresponding assemblages were no different in taxonomic diversity or richness. Targeted lionfish removals reduce lionfish populations effectively elsewhere (Frazer et al., 2012; León et al., 2013), and thus were most likely responsible for the lower lionfish biomass and higher prey fish density observed in removal sites compared to non-removal sites in Roatan. We discard that the observed differences emerged from topographic complexity, hard coral cover, or native predator biomass as these were comparable among removal and non-removal sites. Lionfish larvae supply or post recruitment successes were not quantified here. Given the location of the removal and non-removal sites in relation to prevailing currents (Mehrtens et al., 2001), and the distance between them, these factors likely played a negligible role in driving the observed differences. We recommend to cost-effectively extend the spatial scale at which removal regimes are currently sustained by a cooperation of the diving industry and local management authorities.

While there are currently no systematic studies of lionfish removal effectiveness across the invaded region, studies on lionfish control from a few specific locations report variable levels of density reduction. The reduction levels range from 2-fold reduction in biomass in Curacao and Bonaire and 10-fold reduction in density in Little Cayman to no significant reductions in Colombia (Frazer et al., 2012; León et al., 2013; Bayraktarov et al., 2014). In all of these studies removals were conducted by trained volunteers of the community. Differences in reductions and CPUE among these studies may be related to the initial densities of lionfish and the history of removal effort since the CPUE depends on lionfish abundance and decreases exponentially with repeated removals (Frazer et al., 2012). Thus, the frequency of removals and the time of lionfish replenishment are decisive for the effectiveness of removal programmes. Models suggest that removing 35–65% of adult biomass yearly, or 20–30% monthly, is needed to control population (Barbour et al., 2011; Morris, Rice & Shertzer, 2011; Johnston & Purkis, 2015). Lionfish populations that are left uncontrolled in hydrographically connected areas may offset undertaken control efforts (Ahrenholz & Morris, 2010; Johnston & Purkis, 2015). One major concern for population control are mesophotic ecosystems that lie beyond recreational diving limits and are shown to harbour high abundances of lionfish and larger individuals (Aguilar-Perera, Quijano-Puerto & Hernández-Landa, 2016). In our study, mortality due to removals corresponds to a monthly reduction of ∼10% compared to densities at unfished sites. We argue that repeated removals (two to three times a month) were sufficient to offset the potential replenishment by lionfish adults deriving from unfished sites or depth refugia based on low CPUE and reduced average body sizes of lionfish. Reduced average size resulted from the effect of fishing on population structure, and thus provide further evidence that culling caused the observed differences between removal and non-removal sites. Another study found that lionfish densities on Little Cayman did not replenish over 12–30 days and remained low for more than 70 days once reduced by monthly removals over seven months (Frazer et al., 2012). A reason for this could be that lionfish commonly have high site fidelity and move relatively little (<400 m in diameter) (Bacheler et al., 2015; Tamburello & Côté, 2015). Recolonization by lionfish on reefs that are cleared only once is likely to happen in less than one year (Dahl, Patterson & Snyder, 2016). Although no repeated measurements were taken during this study, personal observations and observations of recreational divers indicate that populations at regular visited sites remained low for at least six months.

The ecological impacts of the lionfish invasion have been widely documented. Invasive lionfish, for instance, exert predation rates on native fishes that exceed those exerted by native predators (Côté & Maljkovic, 2010; Green, Akins & Côté, 2011; Albins, 2013). As a result, lionfish likely severely influence dynamics of prey biomass, particularly at sites where they have become the dominant predators (Green et al., 2014; Albins, 2015; Benkwitt, 2015). In the Bahamas, invasive lionfish caused a density-independent increase in mortality leading to a lionfish-induced extirpation of fairy basslets (*Gramma loreto*) (Ingeman, 2016). Vulnerable populations could thus face an elevated risk of severe reductions or even extinctions, if initiatives to reduce lionfish populations are lacking. Our findings suggest that the observed differences in prey fish density stem from the continuous and frequent removals of lionfish at removal sites. This would support findings of Green et al. (2014) showing that lionfish below densities at which they over-consume prey can effectively prevent declines in prey fish biomass.

One caveat of the study is that study sites were spatially clustered raising the possibility that local variations in variables other than lionfish removal could be responsible for the differences in prey fish density and lionfish biomass. Several factors can influence native coral reef fish assemblages and lionfish such as predation and availability of refuge (Hixon & Beets, 1993; Beukers & Jones, 1998; Bejarano et al., 2015), food availability (Jones, 1986; Forrester, 1990), physical oceanographic processes affecting larval transport (Caselle & Warner, 1996; Ahrenholz & Morris, 2010), as well as recruitment and immigration from surrounding areas (Lewis, 1997; Ault & Johnson, 1998). In our study we accounted for native predator biomass, availability of refugia and hard coral cover. A wider variance around the average native predator biomass occurred in non-removal sites compared to removal sites. This might also explain the lower native prey densities recorded at non-removal sites compared to removal sites. However, all site characteristics were not significantly different between removal and non-removal sites, and are therefore unlikely to have driven the observed differences. The potential influence of larval supply and food availability is relatively difficult to estimate since it depends on the taxon (Valles, Hunte & Kramer, 2009), and on small-scale physical oceanographic patterns that are not well known for Roatan. Lionfish larvae can disperse up to several kilometres depending on strength and direction of ocean currents (Ahrenholz & Morris, 2010; Johnston & Purkis, 2011). Given that our study sites are only a maximum of 3 km apart, facing the same wind exposure (Valdivia et al., 2014) and not separated by a physical barrier, it is unlikely that they differ in lionfish larval supply. Future studies comparing removal and non-removal sites, should consider their potential differences in prey fish larvae supply and recruitment. Post-settlement movements of lionfish to avoid areas where divers are culling regularly may have played a role in observed differences in lionfish biomass because lionfish have been hunted at removal sites for several years (Côté et al., 2014). Potential avoidance behaviour (i.e. quick flight into crevices) by lionfish might also make the detection of lionfish more difficult on removal sites thus counts appear lower (Côté et al., 2014). However maximum recorded movements (1.35 km) of lionfish are still less than distances between removal and non-removal sites (Tamburello & Côté, 2015). Arguably, dive sites could have been originally selected by dive operators based on their higher native fish abundance. If that was the case, site selection by dive operators could have driven the higher abundance of native fishes observed in removal sites. Extensive communication with recreational dive operators, however, indicated that proximity of the reefs to dive centres, cost of fuel for transport, and suitability of dive sites for dive training activities play a stronger role in site selection compared to fish abundance. The most desirable way to study effects of removals onto prey fish communities would have been through before-after-control-impact (BACI) and a higher number of study sites which was not possible in this study. However, studies comparing current situations in removal and non-removal sites on a continuous reef and with naturally occurring lionfish densities remain informative (Elise et al., 2015; Hackerott et al., 2017).

We expected that differences in abundance of prey fish between removal and non-removal sites would correspond to differences in taxonomic richness and diversity. In fact, species richness may decrease proportionately with abundance reductions, as rare species are extirpated by chance (Albins, 2015; Benkwitt, 2015). Consequently, lionfish predation may be more detrimental to species that are endangered or endemic, such as the critically endangered social wrasse *Halichoeres socialis* in Belize, to which lionfish predation is considered a major threat (Rocha et al., 2015). However, we found no significant differences between removal and non-removal sites in taxonomic diversity or richness of native prey. A reason for the lack of difference may be that lionfish are generalists, and this feeding strategy limits the effects of predation on the biomass of one species in particular (Mizrahi et al., 2017). Alternatively, results may reflect that lionfishes feed disproportionally on the most abundant species (Paine, 1966; Stier et al., 2017). A caveat of the study is that we excluded Blennidae and Gobiidae from our censuses. These families have been heavily affected by lionfish predation in other studies (Albins, 2013; Benkwitt, 2015). Further, species richness and diversity is the most stable parameter in a reef fish assemblage which makes it difficult to detect weak differences with a relatively low number of sites surveyed (Kulbicki et al., 2007). Lionfish densities recorded were comparable to other studies that found no effects of lionfish on species richness or diversity (Elise et al., 2015; Hackerott et al., 2017). Importantly, the fact that taxonomic diversity was presumably unaffected by high lionfish biomass is not necessarily an encouraging finding. If a predator preferentially consumes a numerically dominant species, it may cause an increase in taxonomic diversity of prey. This may however imply a reduction in the number of fish that carry out a similar ecosystem function. Further studies are needed to quantify the influence of lionfish predation on the functional diversity of prey based on important functional traits. Considering traits that are informative of the species' trophic roles, or their vulnerability to fishing, would allow for the detection of more profound ecosystem or economic effects of lionfish predation.

Our findings show that the local opportunistic removal regime based on volunteers represents a cost-effective option that may reduce the impact of lionfish considerably. However, removals sustained primarily by volunteers are subject to popular dive sites and depend on willingness. Importantly, no removals take place at sites that are >3 km away from popular recreational dive sites. Given that lionfish larvae are pelagic and can disperse for long distances (Ahrenholz & Morris, 2010), sites without targeted removals may serve as sinks for lionfish replenishment. Removal efforts are concentrated in areas with the highest diving frequency, not necessarily the highest ecological importance. Substantial reductions of lionfish abundance require a long-term commitment and may only be feasible in areas where widespread targeted removals are sustained for multiple consecutive years (Barbour et al., 2011). It is therefore recommended to allocate funds from licensing workshops to support a dedicated and experienced team that regularly hunts at key areas such as spawning aggregation sites and vulnerable nursery sites. A potential strategy might be for dive shops and individual divers to adopt a reef site and visit it regularly. To expand the spatial scale of removal efforts, regular lionfish tournaments can be organised that target areas outside the recreational diving tourism range. A fair and lucrative market for lionfish meat should be developed in order to incentivise fishers to regularly fish on lionfish in other areas of Roatan (Chapman et al., 2016).

## Supplemental Information

10.7717/peerj.3818/supp-1 Supplemental Information 1 Table of raw data.

10.7717/peerj.3818/supp-2 Supplemental Information 2 Supplemental Material. Tables S1 and S2.

## Additional Information and Declarations

### Data Availability

The following information was supplied regarding data availability: The raw data has been supplied as Supplemental Dataset Files.
