# Supplementary material for: Ongoing removals of invasive lionfish in Honduras and their effect on native Caribbean prey fishes
Source: PeerJ. 2017 Oct 18;5:e3818. doi: 10.7717/peerj.3818 (PMC5650727; doi:10.7717/peerj.3818)
Supplement: Supplemental Information 2 — Tables S1 and S2. [file peerj-05-3818-s002.docx]

**Supplements**

**Supplement 1:** Species lists of native predator and native prey fish

Table S1: Native predator species

| **Family** | **Sepcies name** | **Common name** |
| --- | --- | --- |
| **Lutjanidae** | *Lutjanus jocu* | Dog snapper |
|  | *Lutjanus analis* | Mutton snapper |
|  | *Lutjanus apodus* | Schoolmaster |
|  | *Ocyurus chrysurus* | Yellowtail snapper |
| **Serranidae** | *Epinephelus striatus* | Nassau Grouper |
|  | *Mycteroperca tigris* | Tiger Grouper |
|  | *Mycteroperca bonaci* | Black Grouper |
|  | *Mycteroperca interstitialis* | Yellowmouth Grouper |
|  | *Mycteroperca venenosa* | Yellowfin Grouper |

Table S2: Prey fish species

| **Family** | **Scientific name** | **Common name** |
| --- | --- | --- |
| **Acanthuridae** | *Acanthurus bahianus* | Blue Tang |
|  | *Acanthurus coeruleus* | Ocean Surgeonfish |
| **Chaetodontidae** | *Chaetodon capistratus* | Foureye Butterflyfish |
|  | *Chaetodon striatus* | Banded Butterflyfish |
|  | *Chaetodon ocellatus* | Spotfin Butterflyfish |
|  | *Chaetodon sedentarius* | Reef Butterflyfish |
| **Grammatide** | *Gramma loreto* | Fairy Basslet |
| **Hypoplectrus** | *Hypoplectrus indigo* | Indigo Hamlet |
|  | *Hypoplectrus nigricans* | Black Hamlet |
|  | *Hypoplectrus puella* | Barred Hamlet |
| **Labriidae** | *Clepticus parrae* | Creole wrasse |
|  | *Halichoeres garnoti* | Yellowhead wrasse |
|  | *Halichoeres maculipinna* | Clown wrasse |
|  | *Thalassoma bifasciatum* | Bluehead wrasse |
| **Pomacentride** | *Abudefduf saxatilis* | Sergeant Major |
|  | *Chromis cyanea* | Blue Chromis |
|  | *Chromis insolata* | Sunshine fish |
|  | *Chromis multilineata* | Brown Chromis |
|  | *Microspathodon chrysurus* | Yellowtail Damselfish |
|  | *Stegastes adustus* | Dusky Damselfish |
|  | *Stegastes leucostictus* | Beaugregory |
|  | *Stegastes partitus* | Bicolor Damselfish |
|  | *Stegastes planifrons* | Threespot Damselfish |
|  | *Stegastes variabilis* | Cocoa Damselfish |
| **Scaridae** | *Scarus iserti* | Striped Parrotfish |
|  | *Scarus taeniopterus* | Princess Parrotfish |
|  | *Sparisoma aurofrenatum* | Redband Parrotfish |
|  | *Sparisoma viride* | Stoplight Parrotfish |
| **Tetraodontidae** | *Canthigaster rostrata* | Sharpenose Pufferfish |
